# Unlocking the Secrets of the Endometrium: Stem Cells, Niches and Modern Methodologies

**DOI:** 10.3390/biomedicines13102435

**Published:** 2025-10-06

**Authors:** Lijun Huang, Miaoxian Ou, Dunjin Chen, Shuang Zhang

**Affiliations:** 1 Department of Obstetrics and Gynecology, The Third Affiliated Hospital of Guangzhou Medical University, Guangzhou 510150, China; huanglijun94@stu.gzhmu.edu.cn (L.H.); drmiaomx@163.com (M.O.); gzdrchen@gzhmu.edu.cn (D.C.); 2 Key Laboratory for Major Obstetric Diseases of Guangdong Province, Guangzhou 510150, China; 3 Guangdong-Hong Kong-Macao Greater Bay Area Higher Education Joint Laboratory of Maternal-Fetal Medicine, Guangzhou 510150, China; 4 Guangdong Engineering and Technology Research Center of Maternal-Fetal Medicine, Guangzhou 510150, China

**Keywords:** endometrium, endometrial stem cells, organoids, model

## Abstract

The endometrium is a highly dynamic tissue central to female reproductive function, undergoing nearly 500 cycles of proliferation, differentiation, shedding, and regeneration throughout a woman’s reproductive life. This remarkable regenerative capacity is driven by a reservoir of endometrial stem/progenitor cells (ESCs), which are crucial for maintaining tissue homeostasis. Dysregulation of these cells is linked to a variety of clinical disorders, including menstrual abnormalities, infertility, recurrent pregnancy loss, and serious gynecological conditions such as endometriosis and endometrial cancer. Recent advancements in organoid technology and lineage-tracing models have provided insights into the complex cellular hierarchy that underlies endometrial regeneration and differentiation. This review highlights the latest breakthroughs in endometrial stem cell biology, focusing particularly on 3D in vitro platforms that replicate endometrial physiology and disease states. By integrating these cutting-edge approaches, we aim to offer new perspectives on the pathogenesis of endometrial disorders and establish a comprehensive framework for developing precision regenerative therapies.

## 1. Introduction

The human endometrium is a highly dynamic, hormone-responsive mucosal tissue that undergoes approximately 400–500 cycles of cyclical breakdown and scarless regeneration throughout a woman’s reproductive life [1]. This remarkable regenerative capacity is tightly orchestrated by fluctuating levels of estrogen and progesterone, which regulate structural remodeling across the menstrual, proliferative, and secretory phases. These changes are essential for preparing the endometrium to support embryo implantation and pregnancy or to initiate menstruation when fertilization does not occur [2,3]. Traditionally, cyclical regeneration has been attributed to hormone-driven proliferation; however, accumulating evidence underscores the pivotal role of tissue-resident endometrial stem/progenitor cells (ESCs) in driving endometrial repair and regeneration [4,5]. Located primarily within the basalis layer, these ESCs possess self-renewal and multipotent differentiation capabilities, sustaining epithelial and stromal homeostasis after menstruation, delivery, or injury. Disruptions in their function are increasingly linked to endometrial disorders such as endometriosis, implantation failure, intrauterine adhesions, and endometrial cancer [6].

Despite substantial progress in identifying epithelial (e.g., SSEA-1^+^, AXIN2^+^, LGR5^+^) and mesenchymal (e.g., CD140b^+^CD146^+^) progenitor subsets, the hierarchical relationships and spatiotemporal dynamics of these populations remain incompletely defined. One major bottleneck is the lack of physiologically relevant in vitro platforms to model endometrial complexity. While conventional 2D cultures provide limited mechanistic insights, recent advances in 3D organoids, assembloids, and microfluidic “organ-on-chip” systems now enable recapitulation of the endometrium’s architecture, multicellular interactions, and hormone responsiveness. When coupled with single-cell transcriptomics and lineage-tracing models, these systems allow high-resolution interrogation of regeneration, implantation, and disease pathogenesis.

This review synthesizes current knowledge on human endometrial stem/progenitor cells, their niche dynamics, and how advanced in vitro models illuminate the mechanisms of regeneration and clinical dysfunction, paving the way for regenerative and precision therapies in reproductive medicine.

## 2. Endometrial Stem/Progenitor Cells in Endometrial Repair and Regeneration

The human endometrium undergoes cyclical regeneration in synchrony with ovarian steroid hormone dynamics, orchestrated over a canonical 28-day menstrual cycle composed of three distinct yet interdependent phases: menstrual, proliferative, and secretory [7]. The menstrual phase (days 1–4) initiates with the involution of the corpus luteum, leading to progesterone withdrawal. This precipitates ischemic constriction of spiral arterioles and caspase-mediated apoptosis of the functionalis, culminating in extensive shedding of epithelial, stromal, and endothelial compartments [8]. During the proliferative phase (days 5–14), rising levels of 17β-estradiol (E2) drive robust tissue regeneration. This includes polarized expansion of glandular epithelium with lumen formation, stromal thickening via epithelial–mesenchymal transition (EMT), and coordinated angiogenesis and extracellular matrix (ECM) remodeling—ultimately restoring endometrial thickness to approximately 8–12 mm. At mid-cycle (day 14), the luteinizing hormone (LH) surge triggers ovulation and corpus luteum formation, transitioning the endometrium into the progesterone-dominant secretory phase [9]. This period features sequential events: upregulation of implantation markers (e.g., HOXA10, integrin αvβ3), decidual transformation of perivascular stromal cells (characterized by PRL and IGFBP1 expression), and epithelial maturation into secretory and ciliated subtypes. The late secretory phase (days 25–28) is marked by immune cell infiltration—particularly uterine NK cells, macrophages, and regulatory T cells—and prostaglandin-mediated vasoconstriction of spiral arterioles. In the absence of implantation, hormonal support ceases, reactivating apoptotic cascades that drive functionalis breakdown and menstruation [10,11].

Remarkably, tissue shedding and regeneration are not temporally separated but occur concurrently, reflecting the endometrium’s unique regenerative plasticity [12]. Scanning electron microscopy of hysterectomy specimens reveals that re-epithelialization begins as early as 48 h post-menstruation through centripetal migration of residual basal glandular epithelial cells, with full barrier restoration typically achieved by days 5–6 of the cycle [13]. This highly efficient, scarless repair is not limited to menstruation but extends to pregnancy-related processes. Embryo implantation initiates a sequence of remodeling events [14], including embryonic apposition and adhesion, trophoblast invasion with matrix metalloproteinase (MMP)-mediated basement membrane degradation, and progesterone-induced decidual transformation of stromal fibroblasts via transcription factors such as FOXO1 and STAT3 [15]. These changes culminate in the formation of a fetomaternal interface and hemochorial placenta, enabling efficient maternal–fetal exchange through deep trophoblast penetration into the upper myometrium [16]. The uterus displays remarkable adaptability, expanding more than 20-fold to accommodate the developing fetus [17]. Following delivery, postpartum uterine involution mirrors post-traumatic tissue repair, characterized by caspase-dependent apoptosis of hypertrophic cells, macrophage-mediated clearance, and progressive reduction in uterine volume to pregravid levels within six weeks. Importantly, epithelial regeneration begins within 48 h postpartum, with 80% re-epithelialization of non-placental areas achieved by day 7 and complete restoration by day 14. Uterine receptivity is typically reinstated by 40 to 45 days postpartum [18,19]. Collectively, these cyclical and injury-associated regenerative events underscore the endometrium’s unparalleled capacity for tissue remodeling, a property increasingly attributed to resident endometrial stem/progenitor cells (ESCs) [20,21].

In this context, mounting evidence has identified eSPCs as key orchestrators of endometrial renewal. These cells possess both self-renewal ability and multilineage differentiation potential, enabling the regeneration of epithelial and stromal compartments after physiological or pathological insult [22,23]. Two major subtypes have been functionally validated: endometrial epithelial stem cells [24] and endometrial stromal mesenchymal stem cells (EMSCs) [25]. Epithelial stem cells, marked by SSEA-1, AXIN2, and N-cadherin, are primarily located in the basal glands and contribute to luminal and glandular epithelial replenishment during menstruation [26]. Intriguingly, they also exhibit clonal expansion in disease states such as endometriosis and endometrial carcinoma, implicating their dysregulation in hyperplastic disorders. In parallel, EMSCs reside in the perivascular niche and express CD146, PDGFR-β, and SUSD2, playing essential roles in stromal regeneration through paracrine signaling, extracellular matrix modulation, and possibly mesenchymal–epithelial transition [10]. Both stem cell populations operate within specialized microenvironments regulated by ovarian hormones and local niche signals, including Wnt/β-catenin, Notch and mechanical cues [27,28]. Their coordinated activation and differentiation are fundamental for maintaining homeostasis and enabling the remarkable regenerative plasticity that defines the human endometrium.

### 2.1. Dynamic Changes of Epithelial, Stromal, and Immune Cells in Normal Endometrium and Related Diseases

The identification of epithelial stem cells has advanced our understanding of endometrial regeneration and revealed their involvement in proliferative disorders such as endometriosis, endometrial carcinoma, adenomyosis, and intrauterine adhesion (IUA) [10,26,29]. Endometriosis, affecting ~10% of reproductive-age women, is characterized by chronic pelvic pain, infertility, and reduced quality of life [30]. Aberrant stem/progenitor activity in ectopic lesions disrupts cyclic regeneration, leading to glandular disorganization, stromal fibrosis, and chronic inflammation [31].

Recent advancement in single-cell transcriptomics have identified stem-like epithelial (LGR5^+^, SOX9^+^) and stromal populations that establish pro-inflammatory and pro-fibrotic microenvironments [29,31,32]. In this pathological context, stem/progenitor cells that normally sustain repair are hijacked, while immune dysregulation amplifies disease persistence: T and B cells drive chronic inflammation, mast cells recruited via stem cell factor reinforce inflammatory circuits, neutrophils and eosinophils infiltrate in a hormone-dependent manner to support lesion initiation, macrophages (particularly M2 subsets) promote fibrosis and angiogenesis, and impaired NK activity reduces immune clearance [33,34]. Collectively, these immune–stromal–epithelial interactions shape progenitor behavior and sustain the chronicity and invasiveness of endometriosis.

Similar mechanisms may underline malignant transformation. Unlike ectopic lesions that remain histologically benign, endometrial carcinoma arises when progenitor populations acquire oncogenic alterations that subvert their regenerative programs [10]. Lineage-tracing studies have shown that *Axin2*^+^ epithelial stem cells, which normally maintain glandular homeostasis, can initiate tumorigenesis when mutated in pathways such as Wnt/β-catenin, PTEN/PI3K-AKT, or p53 [35]. Single-cell profiling further reveals stem-like subpopulations with heightened plasticity, capable of engaging in EMT/MET cycles that not only promote invasive behavior but also confer therapeutic resistance [36]. Recognizing this dynamic EMT–MET equilibrium is therefore critical to understanding how endometrial stem/progenitor cells maintain physiological plasticity in repair, yet when dysregulated, fuel the progression of benign proliferative disorders and malignant transformation [37].

Together, these findings suggest a continuum whereby endometrial stem/progenitor cells that fuel physiological regeneration may, when dysregulated, first contribute to benign proliferative disorders such as endometriosis and, under cumulative genetic or epigenetic stress, to malignant transformation in endometrial carcinoma. This shared stem-cell axis provides a unifying framework for diverse endometrial pathologies and highlights stemness-associated pathways as promising diagnostic markers and therapeutic targets.

### 2.2. Endometrial Epithelial Stem Cells

**Figure 1:**
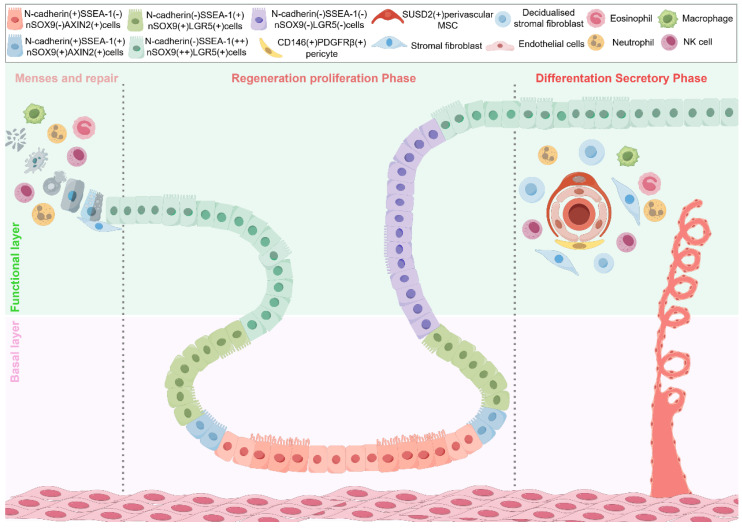
Illustration of the positional hierarchy of endometrial epithelial stem cells in humans during the premenstrual, menstrual/repair, and regenerative phases. Spatial hierarchy of endometrial epithelial stem cells across the menstrual cycle. This illustration depicts the positional hierarchy of endometrial epithelial stem cells in humans during the premenstrual, menstrual/repair, and regenerative phases. It marks the two architectural compartments—the basal layer and the functional layer—and highlights marker-defined epithelial subpopulations (e.g., N-cadherin(+), SSEA-1(−), nSOX9(−), AXIN2(+), among others.

Transitioning from anatomical organization to cellular identity, the identification of definitive epithelial stem cell markers remains a central challenge in endometrial biology. The endometrial glandular epithelium exhibits a unique architectural arrangement: functional glands extend vertically from the luminal surface toward the endometrial–myometrial junction, while their basal horizontal branches form intricate anastomosing networks near the myometrium [38,39]. This spatial configuration closely resembles the crypt–villus axis of the intestine, where blind-ended glandular termini are hypothesized to function as stem cell reservoirs, maintaining a self-renewal gradient along the glandular axis [40]. Advanced three-dimensional reconstructions of full-thickness endometrial tissue using light-sheet fluorescence microscopy have revealed that basal horizontal glands form a connected plexus with direct continuity to vertical glands. Notably, these basal gland remnants are preserved during menstruation. Residual epithelial cells within this region rapidly proliferate and transdifferentiate into flattened cuboidal epithelium, achieving complete luminal coverage by cycle day 5 [41]. This basal niche likely provides dual protection: physical sequestration from mechanical disruption and a specialized microenvironment that sustains stemness and regenerative potential [13,42]. This basal niche likely provides dual protection: physical sequestration from mechanical disruption and a specialized microenvironment that sustains stemness and regenerative potential. In the following section, we summarize the currently proposed candidate markers for epithelial stem/progenitor cells (Figure 1).

#### 2.2.1. SSEA-1

Building on the architectural insights into endometrial glands, SSEA-1 (stage-specific embryonic antigen-1) was the first functionally validated marker for epithelial progenitors. Initial clonogenic assays revealed that only 0.22% of epithelial cells formed large colonies with high proliferative capacity [43]. Culture conditions optimized with TGFα, EGF, and PDGF-BB increased colony-forming efficiency 3.2-fold, highlighting the reliance of these progenitors on growth factor signaling [43,44]. SSEA-1^+^ cells exhibit hormonal responsiveness, with elevated abundance during the proliferative phase in correlation with serum estradiol levels. These cells can be serially passaged in vitro and differentiate into both glandular and luminal epithelial lineages, confirming their progenitor identity [45]. Improved culture systems employing small-molecule cocktails now allow long-term expansion of SSEA-1^+^ populations, advancing their therapeutic potential. In intrauterine adhesion models, SSEA-1^+^ cells outperform mesenchymal stem cells by reducing fibrosis and enhancing regeneration [24] SSEA-1^+^ spheroids exhibit nuclear β-catenin accumulation but minimal expression of ERα and PR, suggesting active Wnt signaling maintains their undifferentiated state [46].

#### 2.2.2. N-Cadherin

Though overlapping with SSEA-1^+^ cells spatially, N-cadherin marks a predominantly quiescent subset, suggesting a hierarchical organization regulated by Wnt gradients.

Complementing SSEA-1, N-cadherin (CDH2)—a canonical Wnt/β-catenin target—marks a distinct epithelial progenitor subpopulation. A Wnt signaling gradient from the basalis to the luminal surface was shown to regulate stem cell activation. N-cadherin^+^ epithelial cells display superior clonogenic capacity and form CK^+^ gland-like structures in 3D culture [47]. Immunohistochemistry localizes these cells to the basal region of endometrial glands, co-expressing epithelial markers such as E-cadherin and ERα while lacking mesenchymal traits. These cells remain largely quiescent under homeostatic conditions, with minimal Ki67 expression [48]. Advanced 3D glandular reconstruction [22] and tissue-clearing [49] confirm that N-cadherin^+^ cells anchor a root-like network within the basalis.

While SOX9^+^SSEA-1^+^ cells are shed with the functionalis during menstruation, lineage-tracing studies reveal that the true regenerative reservoir resides at the interface between the basalis and functionalis. At this junction, SSEA-1^+^SOX9^+^ cells transiently persist during the early stages of re-epithelialization before committing to terminal differentiation [50]. Despite partial spatial overlap with SSEA-1^+^ cells in the basalis, N-cadherin marks a phenotypically distinct subset characterized by relative quiescence. Whereas SSEA-1 labels actively cycling progenitors engaged in tissue regeneration, N-cadherin identifies a largely dormant population, suggesting the existence of a hierarchical organization within the epithelial stem cell compartment. This spatial and functional heterogeneity implies that Wnt signaling gradients may orchestrate the dynamic balance between progenitor activation and dormancy.

#### 2.2.3. AXIN2

AXIN2, a dual-function regulator of the Wnt signaling pathway and a lineage-tracing marker, plays a pivotal role in defining the hierarchy of endometrial epithelial progenitors. Its subcellular localization dictates Wnt pathway dynamics: nuclear AXIN2 promotes β-catenin degradation and suppresses Wnt activity, whereas cytoplasmic AXIN2 facilitates β-catenin stabilization and pathway activation [47]. Pulse-chase labeling studies have demonstrated that epithelial renewal is predominantly driven by *Axin2^+^* progenitor cells rather than by differentiated epithelial cells. In *Axin2^rtTA^*; *tetO^H2BGFP^* mice, lineage-tracing experiments reveal that *Axin2^+^* cells are localized at the base of endometrial glands and give rise to both luminal and glandular epithelial lineages across multiple estrous cycles. Functional validation using 3D organoid models confirms that only *Axin2^+^* cells are capable of initiating fully polarized gland-like structures, whereas *Axin2^−^* cells fail to form organoids. Targeted ablation of this population in *Axin2^rtTA^*; *tetO^Cre^; R26-iDTR* mice results in complete glandular collapse and cessation of luminal epithelial proliferation, underscoring their indispensable role in endometrial homeostasis. Furthermore, *Axin2*^+^ cells co-express canonical stem/progenitor markers such as *Lgr5* and *Trop2*, and are uniquely susceptible to oncogenic transformation. In genetically engineered mouse models harboring *Axin2*^+^-specific mutations (*Axin2^rtTA^*; *tetO^Cre^*; *R26-Pik3ca*, *EGFP^f/+^*; *Ctnnb1^f(ex3)/+^*), these cells serve as the cell of origin for endometrial adenocarcinoma, giving rise to tumors that recapitulate key features of human pathology [35]. Collectively, these findings establish *Axin2*^+^ cells as long-lived, bipotent epithelial progenitors that govern both physiological regeneration and malignant transformation in the endometrium.

#### 2.2.4. SOX9 (nSOX9)

SOX9 functions as a master transcriptional integrator bridging Wnt signaling with epithelial stem/progenitor cell regulation in the endometrium [51]. As a member of the SRY-box (SOX) transcription factor family, it exhibits phase-specific expression patterns—peaking during the estrogen-dominant proliferative phase and declining during the secretory phase—paralleling cyclical progenitor activation [52]. Sustained overexpression of SOX9 has been linked to endometrial carcinogenesis, whereas its physiologic oscillation supports progenitor maintenance. In pathologic states such as endometriosis, SOX9 dynamics become disrupted; in 3D culture, ectopic epithelial cells self-organize into polarized gland-like structures displaying apical–basal SOX9 gradients, implicating its role in morphogenetic regulation [53].

Integrative single-cell RNA sequencing and spatial transcriptomics have recently mapped SOX9^+^ populations across luminal, glandular, and basal compartments of the endometrium [8,28,54]. Garcia et al. identified a discrete SOX9^+^ epithelial cluster from 98,568 uterine cells, revealing three anatomically distinct subtypes [54], (1) noncycling SOX9^+^LGR5^+^ cells enriched in the luminal epithelium; (2) noncycling SOX9^+^LGR5^−^ cells localized to basal glands; and (3) cycling SOX9^+^ cells within superficial regenerating glands. This spatial organization is hormonally regulated—SOX9 expression peaks during proliferative phases, supporting epithelial renewal, and declines during secretory phases as cells adopt secretory differentiation fates.

Recent 2024 updates further refined the spatial hierarchy of SOX9^+^ populations [28], two principal compartments were defined: Basalis SOX9^+^ cells, anatomically restricted to the basal glands, co-express canonical progenitor markers including N-cadherin, AXIN2, and ALDH1A1. Cell–cell interaction analysis highlighted signaling hubs involving CXCL12, WNT and FGF pathways—hallmarks of niche-regulated stem cell maintenance. SOX9 functionalis I and II were mapped to the functionalis glands. Functionalis I had high SOX9 and CDH2 expression, marked by PHLDA1/SLC7A11 and DKK1-mediated AXIN2 suppression, and functionalis II had lower SOX9/CDH2 levels, characterized by KMO, IHH, and EMID1 enrichment.

In parallel, proliferative-phase luminal epithelium harbors cycling SOX9^+^LGR5^+^ cells coexisting with pre-ciliated and ciliated lineages, consistent with established epithelial maturation trajectories [28]. These findings collectively position SOX9^+^ cells within a spatially and temporally dynamic hierarchy, coordinating endometrial regeneration, differentiation, and hormonal responsiveness—mirroring the structural and functional logic described for SSEA-1^+^, N-cadherin^+^, and Axin2^+^ epithelial progenitors.

#### 2.2.5. LGR5

LGR5, a transmembrane receptor for R-spondins, enhances Wnt/β-catenin signaling by forming ternary complexes with Frizzled and LRP receptors [55]. It is widely recognized as a conserved marker of adult stem cells across multiple tissues, including the intestine, stomach, liver, ovaries, and fallopian tubes [56,57]. However, its expression and function in the endometrium diverge significantly from these canonical roles. In human endometrial tissue, LGR5 expression is predominantly restricted to the luminal epithelium, with minimal overlap with established epithelial progenitor markers such as SSEA-1 and SOX9. This non-glandular localization raises questions about its role as a true epithelial stem/progenitor marker within the endometrial context. Technical challenges further complicate its interpretation—particularly antibody cross-reactivity with hematopoietic cell markers (e.g., CD45, CD163), which may confound the identification of bona fide epithelial LGR5^+^ populations in human samples [58]. These issues, along with its spatial discordance from known regenerative niches, have led to ongoing debate regarding the validity of LGR5 as a definitive marker for endometrial stem/progenitor cells.

Insights from murine models offer partial resolution. Using the *Lgr5-2A-CreERT2; R26-tdTomato* lineage-tracing system, Seishima et al. demonstrated that during early postnatal glandulogenesis, *Lgr5^+^* cells contribute to both luminal epithelium and glandular buds, consistent with a bipotent progenitor role [59]. However, when tracing was initiated after gland formation, *Lgr5^+^* cells selectively labeled the glandular epithelium (GE), with no significant contribution to the luminal compartment. Functional studies using *LGR5-DTR-EGFP* ablation confirmed this restriction—targeted depletion of *Lgr5^+^* cells impaired GE homeostasis without affecting the luminal epithelium (LE) [59]. These findings suggest a temporal transition in Lgr5 function: from a bipotent progenitor pool during organogenesis to a more lineage-restricted stem cell population in adulthood. Notably, *Lgr5* expression declines after puberty, further supporting this developmental restriction. Despite these insights, the lack of conserved expression patterns and unresolved technical issues in human endometrial tissue underscore the need for species-specific validation. Thus, while *Lgr5^+^* cells contribute to adult glandular maintenance in mice, their relevance to human endometrial regeneration remains uncertain and warrants further investigation [58].

#### 2.2.6. “Junctional Zone” Stem Cell

The discovery of a “junctional zone” stem/progenitor cell population by Jin et al. has redefined existing paradigms of endometrial regeneration [60]. Using *Krt19-CreERT2; R26-Tomato* mice and single-cell pulse-chase lineage tracing, the authors identified a bipotent epithelial progenitor population residing at the interface between the luminal epithelium (LE) and the glandular epithelium (GE)—termed the junctional zone. These cells exhibited robust regenerative potential, giving rise to both EpCAM^+^Foxa2^−^ luminal and EpCAM^+^Foxa2^+^ glandular lineages. Remarkably, this clonal multilineage capacity persisted across 72 estrous cycles (approximately 360 days), underscoring their long-term self-renewal and bipotency in vivo.

Mechanistic insights were further validated using dual-pulse labeling with *Foxa2-CreERT2; R26-Tomato* mice, which demonstrated a defined differentiation trajectory. During each cycle, junctional zone stem cells generated lineage-committed precursors that followed distinct migratory routes: luminal precursors moved radially to repopulate the surface epithelium, while glandular precursors migrated basally along the gland necks to sustain glandular homeostasis. This spatially and temporally coordinated differentiation program supports synchronized tissue renewal without disturbing endometrial architecture [60]. These findings highlight the junctional zone as a previously unrecognized niche for long-lived bipotent epithelial stem/progenitor cells and provide a mechanistic basis for the precise coordination of cyclical endometrial regeneration.

#### 2.2.7. The Hierarchical Structure of Endometrial Epithelial Stem Cells

The spatially distinct expression patterns of epithelial stem/progenitor markers in the human endometrium point to a hierarchical cellular organization [1,26,61] (Figure 1). At the apex of this hierarchy reside N-cadherin^+^SSEA-1^−^SOX9^−^ cells within the basal gland crypts—quiescent primordial progenitors presumed to serve as the ultimate epithelial reserve. These rare cells intermittently give rise to N-cadherin^+^SSEA-1^+^SOX9^+^ transitional progenitors, which subsequently differentiate into N-cadherin^−^SSEA-1^+^SOX9^+^ junctional progenitors located at the basalis–functionalis interface. This intermediate population expands transiently during the proliferative phase, contributing to epithelial regeneration. Terminal differentiation gives rise to N-cadherin^−^SSEA-1^−^SOX9^−^ glandular epithelial cells, which ascend to populate the luminal epithelium—prone to cyclical shedding and renewal.

This hierarchical model addresses a long-standing paradox in endometrial biology: if regenerative progenitors resided solely within the superficial functionalis or the cycling basalis, repeated monthly shedding would exhaust their proliferative reserve. To resolve this, Jin et al. proposed a dual-reservoir model [60] comprising short-lived progenitors within the functionalis that support rapid, hormone-driven renewal; and long-lived stem cells anchored in the basal gland network, protected within a morphologically stable and signaling-insulated niche. The basalis niche—characterized by dense glandular labyrinths and restricted exposure to cyclical hormonal fluctuations—maintains a regenerative reserve through Wnt/Notch signaling gradients and spatial sequestration. In contrast, functionalis-derived progenitors rapidly proliferate in response to estrogenic cues, enabling efficient turnover while preserving architectural integrity. This division of labor ensures both physiological regeneration and long-term tissue fidelity.

### 2.3. Endometrial Mesenchymal Stem Cells (eMSCs)

While epithelial stem cells predominantly mediate cyclical re-epithelialization, endometrial mesenchymal stem cells (eMSCs)—localized within the stromal compartment—serve as indispensable counterparts by regulating tissue remodeling, stromal regeneration, and epithelial-stromal crosstalk. The endometrial stroma is composed of heterogeneous cell types, including fibroblasts, immune cells, and a vascular-associated mesenchymal population, which modulate tissue integrity via secretion of niche-supportive signals (e.g., WNTs, FGFs, chemokines), inflammatory regulation, and scarless repair. These properties are particularly evident in regenerative responses following pathological insult, such as intrauterine adhesions and endometriosis.

#### 2.3.1. SUSD2

Initially identified as a rare population (~1.25%) of clonogenic stromal progenitors [43], eMSCs have since been defined by expression of classical MSC markers—CD29, CD44, CD73, CD90, CD105, CD117, CD140b (PDGFRβ), and CD146—with a unique spatial association to perivascular niches [62]. Among these, SUSD2 (W5C5) has emerged as a robust identifier of functionally superior eMSC subsets, exhibiting >3-fold enhanced clonogenicity and multilineage differentiation potential compared to SUSD2^−^ counterparts [63]. These SUSD2^+^ cells generate adipogenic, osteogenic, chondrogenic, myogenic, and endothelial lineages, and form stroma-like tissue in vivo when xenografted beneath the renal capsule of immunodeficient (NSG) mice [62]. Functional assays further validate the stem-like properties of dual-positive CD146^+^PDGFRβ^+^ perivascular cells, which demonstrate significantly higher colony-forming efficiency compared to unselected stromal populations [64]. Collectively, these findings establish SUSD2^+^CD146^+^PDGFRβ^+^ perivascular cells as a principal eMSC reservoir responsible for orchestrating stromal-epithelial crosstalk and cyclic stromal regeneration.

#### 2.3.2. PDGFRα

Cross-species studies have delineated a conserved stromal hierarchy wherein perivascular eMSCs contribute to regeneration via both direct differentiation and paracrine signaling. Spitzer et al. isolated CD146^+^PDGFRβ^+^ perivascular stem cells from human endometrium, demonstrating their multipotency and enrichment for self-renewal and immunomodulatory gene signatures—hallmarks of bona fide mesenchymal stem cells [65]. Building on this, Kirkwood et al. [66] employed scRNA-seq in a murine menstruation model, identifying a transitional PDGFRβ^+^ stromal population co-expressing mesenchymal markers (Vimentin, Pdgfrβ), epithelial markers (EpCAM, Krt18), and MET-associated transcription factors. Lineage tracing with *Pdgfra-creERT2*; *R26-tdTomato* mice confirmed that PDGFRα^+^ fibroblasts undergo mesenchymal-to-epithelial transition (MET) and contribute to regenerated luminal epithelium. Interestingly, PDGFRβ^+^ cells retained hybrid transcriptional states, suggesting partial lineage commitment. These observations suggest a division of labor within the stromal compartment: PDGFRβ^+^ perivascular cells function as transitional progenitors, while PDGFRα^+^ fibroblasts serve as mobile repair effectors, enabling spatially coordinated regeneration during each menstrual cycle [66].

#### 2.3.3. Perivascular eMSCs

Perivascular eMSCs share extensive transcriptional and phenotypic overlap with traditional MSCs [67] and are postulated to represent their cellular origin within the uterus [68]. In addition to SUSD2/CD146/PDGFRβ [63]. markers such as NG2, Stro-1, EphA3, W8B2, CD271, and CD34 delineate perivascular and adventitial subpopulations. However, their functional capacity is not equivalent. For example, CD34^+^ cells fail to regenerate endometrial tissue in xenotransplant models [69], whereas scRNA-seq in mice reveals transient expansion of perivascular cells during repair, followed by their disappearance post-regeneration—implying a repair-specific, non-homeostatic role.

Further complicating this landscape, lineage tracing using *Ng2-CreER* mice shows no contribution of *Ng2*^+^ perivascular cells to epithelial repair [70]. This discrepancy may reflect unrecognized heterogeneity within perivascular compartments, where at least two subsets may coexist: a resident eMSC population and a transient injury-induced population, with Ng2-CreER models potentially labeling only the latter. This underscores the need for combinatorial marker strategies and temporally controlled lineage tracing to fully capture the regenerative spectrum of eMSCs.

#### 2.3.4. Mesenchymal–Epithelial Transition (MET)

The capacity of eMSCs to contribute to endometrial repair via mesenchymal–epithelial transition (MET) remains a subject of ongoing debate. While MET is well-documented during embryonic uterine development and decidualization [71], its role in adult cyclical repair is controversial. Classical histological studies argue that re-epithelialization primarily arises from glandular remnants, wherein proliferative epithelial cells migrate laterally to re-cover denuded surfaces [72,73].

However, lineage tracing in postpartum and menstrual injury models challenges this paradigm [74]. For instance, *Amhr2-Cre*; *Rosa26-EYFP* tracing in menstruation-like injury models identified YFP^+^ transitional stromal cells co-expressing pan-cytokeratin and vimentin, suggesting MET-derived epithelial contributions [70,75]. Likewise, SM22α-Cre-labeled *CD34^+^KLF4^+^* stromal cells were shown to integrate into luminal epithelium following MET in murine models [76]. Yet, Ghosh et al. using *Amhr2-Cre; R26-LacZ* and *Pdgfrβ^rtTA^*; *tetO^Cre^*; *Rosa26-lacZ* mice, observed no stromal-to-epithelial contribution during adult cyclic turnover, and attributed embryonic labeling of epithelial cells to Amhr2-expressing coelomic progenitors, rather than adult MET [77]. To reconcile these inconsistencies, Spooner-Harris et al. demonstrated hormonal modulation of MET in *Amhr2-Cre-YFP/GFP* mice: MET-derived epithelial cells peaked during proestrus, but were absent by diestrus, implying transient hormonal induction rather than steady-state MET [78]. Although these cells were indistinguishable from native epithelium by markers (*EpCAM^+^/FOXA2^+^/ESR1^+^*), they exhibited functional limitations, contributing primarily to glandular niches during development, with limited involvement in cyclic repair [78].

Collectively, these findings suggest that MET occurs in a context-dependent manner, likely restricted to hormonally primed or injury-associated states (e.g., postpartum repair, proestrus phase), rather than playing a central role in routine menstruation. Divergent findings across models may reflect technical variables such as Cre driver specificity, labeling efficiency, timing of tracing, and hormonal milieu, highlighting the urgent need for standardized, temporally synchronized lineage-tracing platforms to define the physiological relevance of MET in endometrial biology [26].

While early studies have successfully identified putative epithelial progenitor markers—such as Axin2^+^, Lgr5^+^, and SOX9^+^ cells—and have elucidated niche signaling pathways involving Wnt, Notch, and hormonal axes, the dynamic interplay between endometrial stem/progenitor cells (ESCs) and their surrounding niche remains incompletely understood. In particular, how N-cadherin^+^ basal glandular progenitors coordinate with adjacent fibroblasts, vasculature, and immune cells during tissue repair and implantation remains a critical area of investigation. The relative contributions of mesenchymal stem cells (MSCs) via paracrine signaling or mesenchymal–epithelial transition (MET) to endometrial regeneration also remain controversial [79]. While mesenchymal-to-epithelial transition (MET) is important for endometrial repair, the reverse process, epithelial-to-mesenchymal transition (EMT), also contributes to regeneration and pathology [36]. Transient EMT facilitates epithelial migration and re-epithelialization during normal remodeling, whereas persistent or dysregulated EMT drives fibrosis, invasion, and therapeutic resistance, particularly in endometriosis and endometrial carcinoma [80,81]. This highlights the need to view EMT and MET as interdependent processes within a dynamic equilibrium.

Recent single-cell multi-omics and spatial transcriptomics analyses have begun to resolve the cellular heterogeneity and niche organization across the menstrual cycle, identifying dynamic populations of CD90^low^ fibroblasts, perivascular cells, and cycling SOX9^+^ progenitors that coordinate with hormonal cues to drive regeneration and decidualization [4]. Importantly, vascular endothelial cells have been shown to secrete VEGF and IL-6 upon injury, initiating localized angiogenesis and immune recruitment [82]. Concurrently, laminin- and fibronectin-rich ECMs secreted by basalis fibroblasts provide essential biomechanical and biochemical scaffolding for progenitor maintenance and activation [83].

## 3. Modeling Endometrial Dynamics and Functionality In Vitro

Comparative studies reveal important species differences in endometrial stem/progenitor biology. Unlike women, mice do not menstruate and rely on induced “menstrual-like” models, where variables such as progesterone withdrawal timing alter repair dynamics and immune responses, potentially confounding stem/progenitor readouts [84]. While lineage-tracing in mice has identified label-retaining stromal and epithelial progenitors, these findings should be viewed as models of endometrial breakdown and repair rather than direct equivalents of human menstruation, highlighting the need for cross-validation in human tissues and organoid systems [85].

In humans, three stem/progenitor compartments are well established, such as perivascular stromal eMSCs (CD146^+^/CD140b^+^ or SUSD2^+^), and epithelial progenitors in the basalis layer (N-cadherin^+^, SSEA-1^+^, SOX9). These populations have been validated by clonogenicity, long-term culture, multilineage differentiation assays and single-cell and spatial transcriptomics, underscoring that not all murine-identified progenitors are conserved in humans.

While in vivo studies have greatly advanced our understanding of endometrial stem/progenitor cell biology, mechanistic dissection of their spatiotemporal behavior, lineage progression, and niche interactions remains challenging due to ethical and technical constraints associated with human endometrial sampling, as well as the limited physiological relevance of non-primate models [86,87,88,89,90,91].

Although 2D cultures have provided key insights into hormone and cytokine signaling, their structural oversimplification—lacking epithelial polarization, stromal-epithelial crosstalk, and tissue-level organization—severely restricts their ability to recapitulate the native endometrial microenvironment [15,91,92,93,94,95].

**Figure 2:**
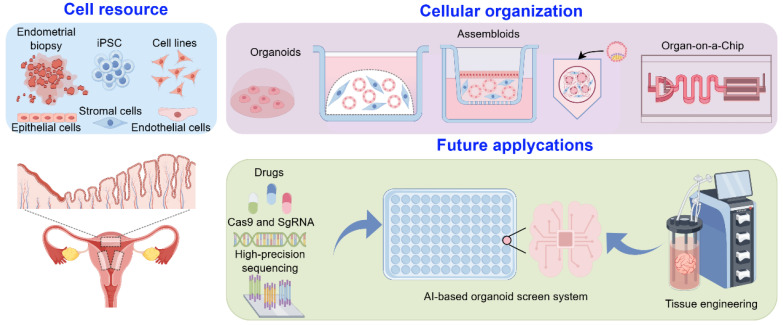
Advanced in vitro platforms modeling human endometrial regeneration and embryo implantation. Left panel: Cell resources for endometrial models, including endometrial biopsy-derived cells, induced pluripotent stem cells (iPSCs), established cell lines, and constituent cell types such as stromal, epithelial, and endothelial cells. Upper right panel: In vitro tissue platforms—from organoids and assembloids to organ-on-a-chip systems—that recapitulate endometrial tissue architecture and function. Lower right panel: Future applications, including drug screening, CRISPR-Cas9 and sgRNA-based gene editing, high-precision sequencing, AI-driven organoid screening systems, and tissue engineering approaches.

To bridge this gap, next-generation 3D culture platforms—including organoids, microfluidic systems, and bioengineered scaffolds—are emerging as physiologically relevant models that recapitulate the architectural and functional complexity of the endometrium (Figure 2). These advanced systems enable spatially resolved hormone signaling, dynamic cell–matrix interactions, and regenerative remodeling, offering unprecedented opportunities to study human-specific embryo implantation and endometrial repair mechanisms.

### 3.1. Organoids

The development of 3D endometrial organoid systems has revolutionized the ability to model endometrial physiology and stem cell behavior in vitro [96]. Initial studies by Bläuer et al. cultured human endometrial epithelial cells in Matrigel overlays with stromal support, demonstrating preserved hormonal responsiveness to estradiol and medroxyprogesterone acetate [97]. A pivotal breakthrough in 2017 enabled the establishment of long-term, expandable human and mouse epithelial organoids using a defined medium containing EGF, Noggin, R-spondin-1, FGF10, HGF, A83-01 (a TGF-β inhibitor), and nicotinamide [98,99]. Under these conditions, single EpCAM^+^ cells self-organize into apicobasally polarized spheroids that mirror in vivo differentiation into secretory and ciliated cell types, recapitulating menstrual-phase dynamics and retaining transcriptomic stability over serial passaging, as confirmed by comparative genomic hybridization [98,99].

Beyond their use as hormonal response models, endometrial organoids have become powerful platforms for dissecting stem/progenitor cell function. Clonal derivation from single epithelial cells allows direct assessment of self-renewal and differentiation. Organoids derived from basalis-enriched tissues display higher clonogenicity and bipotent capacity, reflecting the dominance of progenitor populations such as *Lgr5^+^* and *Axin2^+^* cells in this compartment. In murine studies, lineage tracing of *Axin2^+^* and *Lgr5^+^* epithelial stem cells confirmed their ability to initiate organoid formation and generate both glandular and luminal lineages, validating their stemness in vitro [35]. Similarly, SOX9^+^ human epithelial cells produce long-term expandable organoids responsive to estrogen and progesterone, and the differentiation direction of the SOX9+ cell population is precisely regulated by the WNT and NOTCH signaling pathways [54]. Importantly, patient-derived organoid models from decidua [100,101], menstrual effluent [102], and pathological tissues—including endometriosis, hyperplasia, and endometrial carcinoma—retain disease-specific histopathology, transcriptomic features, and somatic mutations [99,103,104]. These models enable longitudinal study of aberrant progenitor activity, hormone resistance, and treatment response, establishing a translational platform for precision medicine. Moreover, recent advances integrate single-cell transcriptomics and bulk RNA-seq to map intra-organoid heterogeneity and reconstruct in vitro epithelial hierarchies. Fitzgerald et al. demonstrated that donor tissue origin (proliferative vs. secretory phase) and organoid subclone lineage impact hormonal sensitivity, stem cell marker expression, and differentiation dynamics [105]. Standardized hormone-pulse protocols, mechanical stimulation (e.g., hydrostatic pressure), and single-cell guided subcloning now enhance reproducibility and resolution in modeling regenerative cycles [106,107].

In summary, endometrial organoid systems serve as a critical bridge between stem cell identification and functional validation, enabling mechanistic exploration of endometrial regeneration, disease modeling, and personalized therapy development in a human-relevant context.

### 3.2. Assembloids

While epithelial organoids have significantly advanced in vitro modeling of hormone responsiveness and stem/progenitor dynamics, they lack the multicellular complexity and spatial organization of native endometrium. To address these limitations, endometrial assembloids—self-organizing, multi-lineage 3D constructs—have emerged as next-generation platforms that incorporate epithelial, stromal, endothelial, and immune components to better recapitulate tissue architecture and function [108].

Initial efforts utilized human-derived stromal fibroblasts, endothelial cells (e.g., HUVECs), and epithelial cells embedded within hydrogels such as gelatin-methacryloyl (GelMA), modulating stiffness and cell ratios to simulate physiological biomechanical cues [109]. While such configurations enabled vascular network formation and early trophoblast invasion modeling (e.g., HTR-8/SVneo), they often lacked luminal epithelial integrity and failed to reproduce perivascular niche support, limiting their physiological relevance [110].

Cheung et al. [101]. further integrated iPSC-derived stromal fibroblasts (PSC-ESFs) with postpartum epithelial cells, achieving long-term culture with preserved hormonal responsiveness and stromal signaling. However, the model could not sustain co-culture with primary epithelial organoids, underscoring the importance of cell-type compatibility for maintaining epithelial–stromal crosstalk [101].

A major advancement came from Rawlings et al. [111]., who developed assembloids using patient-matched epithelial organoids and stromal fibroblasts. Upon hormonal decidualization, single-cell transcriptomics revealed functionally distinct stromal subpopulations, including a senescent subset that secreted implantation-facilitating factors. Co-culture with human embryos demonstrated enhanced blastocyst adhesion and outgrowth, establishing this system as a robust platform for dissecting maternal–fetal signaling during implantation [111].

Building on this, Tian et al. [112]. introduced a biomechanically optimized air–liquid interface assembloid (ALI-EnAo) by co-culturing primary epithelial and stromal cells in a viscoelastic matrix, exposing the apical surface to air. This configuration restored a ciliated, hormone-responsive luminal epithelium and faithfully recapitulated cyclic transcriptional dynamics across menstrual phases, particularly during the window of implantation (WOI). The ALI system enabled direct embryo access to the epithelium, overcoming limitations of submerged cultures and supporting high-resolution embryo–endometrium interaction studies [112].

In another refinement, uterus-derived extracellular matrix (UEM) was applied as a scaffold enriched in endometrium-specific proteins, notably fibronectin and decorin. UEM significantly improved epithelial regeneration, stromal decidualization, and blastocyst invasion compared to Matrigel. Decorin also upregulated Wnt7a—a critical regulator of endometrial development—enhancing both structural and functional fidelity. This biomimetic scaffold facilitated the generation of hormonally responsive, spatially organized assembloids that closely mimic in vivo implantation conditions, offering a valuable tool for regenerative medicine and mechanistic studies [113].

To further improve multicellular architecture, Shibata et al. [114]. constructed apical-out endometrial organoids (AO-EMOs) embedded in a collagen-enriched matrix and cultured in chemically defined minimal medium. Integration of stromal fibroblasts (eSCs) and HUVECs formed anatomically relevant tissue layers, including polarized epithelium, compact stroma, and self-organized vasculature. Single-cell transcriptomic profiling revealed active epithelial–stromal–endothelial communication (e.g., CXCL12–CXCR4, VEGF–KDR), while co-culture with human blastoids triggered epithelial breach, trophoblast invasion, decidualization, and angiogenesis—closely recapitulating peri-implantation events [114].

Complementing the human model, a murine apical-out endometrial organoid was generated by co-culturing mouse epithelial and stromal cells in 3D low-attachment conditions. These organoids exhibited correct polarity (E-cadherin^+^ epithelium, vimentin^+^ stroma, apical MUC1), responded to sex steroids, and supported key implantation stages—blastocyst attachment, epithelial invagination, entosis, and invasion. Marker analysis confirmed trophoblast differentiation and decidualization (stromal COX-2 expression), establishing this model as a physiomimetic, scalable tool for studying embryo–uterine interactions [115]. Finally, under air–liquid interface culture, authentic mouse blastocysts were integrated with uterine epithelial–stromal modules to generate a high-fidelity implantation model. This system recapitulated >90% implantation efficiency, induced robust maternal COX-2 expression, and activated embryonic AKT signaling at the interface. Notably, AKT1 transduction in embryos rescued COX-2 inhibitor–induced implantation failure in vivo, reinforcing the functional relevance of conserved maternal–embryonic signaling circuits (e.g., COX-2–AKT1) and establishing a tractable platform for developmental and therapeutic studies [116].

These platforms have successfully recapitulated key features of the implantation microenvironment—including hormonal responsiveness, decidual subtypes, epithelial polarity, vascular self-assembly, and embryo–maternal crosstalk—thereby offering physiomimetic systems for dissecting peri-implantation biology. Innovations such as air–liquid interface culture, uterus-derived ECM scaffolds, and apical-out polarity have significantly improved structural and functional fidelity. Cross-species validation in both human and murine models further supports their translational relevance.

### 3.3. Microfluidic Systems (Organ-on-a-Chip)

Despite notable advances, current endometrial assembloids are constrained by static culture conditions, lacking physiological fluid dynamics, mechanical stimulation, and systemic hormone oscillations—features essential for faithfully modeling implantation and cyclic remodeling.

To overcome these limitations, organ-on-a-chip (OoC) platforms have emerged as a powerful extension. These microfluidic-based systems incorporate living cells into architecturally defined and mechanically active microenvironments, enabling real-time simulation of tissue–tissue interfaces, hormonal pulsatility, and shear stress under controlled conditions [117]. Within female reproductive biology, endometrium-on-chip technologies are now positioned at the frontier for modeling menstrual cycle dynamics, embryo implantation, pharmacologic responses, and disease pathogenesis [117,118].

Pioneering work by Gnecco et al. introduced a dual-channel microfluidic chip to model the vascular–stromal interface of the human endometrium [119]. They demonstrated that shear stress from perfused endothelial channels promotes stromal decidualization via flow-responsive paracrine signaling, under hormone-pulsed conditions simulating a 28-day menstrual cycle [119]. Building on this, Ahn et al. developed a vascularized endometrium-on-chip comprising primary epithelial, stromal, and endothelial cells [120]. The model revealed hormone-modulated barrier function and vascular remodeling, although translational relevance was limited by non-physiological hormone levels and non-endometrial endothelial sources [120].

To simulate systemic reproductive endocrine crosstalk, Xiao et al. engineered a multi-organ chip connecting ovary, fallopian tube, endometrium, cervix, and liver [121]. This platform recapitulated key features of the human menstrual cycle, including LH surges and hCG secretion, and enabled pharmacokinetic evaluation of endocrine-disrupting agents (e.g., BPA-induced follicular atresia) [121]. In parallel, Park et al. established a static dual-organ chip integrating endometrial organoids and ovarian follicles within a decellularized uterine ECM hydrogel [122]. This construct preserved viability, steroidogenesis, and epithelial barrier integrity, and predicted teratogenic toxicity of compounds such as thalidomide and valproic acid with high sensitivity [122].

Endometrium-on-chip systems thus offer enhanced physiological fidelity for studying implantation and reproductive toxicology. Yet, challenges remain in standardizing hormonal gradients, validating against clinical phenotypes, and incorporating immunological components. Future directions should focus on integrating patient-specific iPSC lines, CRISPR-based disease modeling, and machine learning–guided chip optimization to support long-term stability, immune–epithelial interaction, and regulatory validation under FDA/EMA frameworks.

Recent advances in stem cell–derived assembloids and organ-on-chip platforms have revolutionized in vitro modeling of the endometrium. Bioengineered assembloids combining epithelial, stromal, endothelial, and immune cells now recapitulate key features such as hormone responsiveness, epithelial polarity, vascularization, decidualization, and embryo implantation. Scaffold innovations—such as uterus-derived extracellular matrix (UEM) [113] and biomechanically tuned air–liquid interface systems—have addressed the limitations of Matrigel, while emerging natural and hybrid hydrogels further preserve endometrium-specific biochemical cues and support more physiologically relevant hormone and immune responses [123,124,125]. Endometrium-on-chip models further enable real-time analysis of shear stress, hormonal pulsatility, and barrier function, advancing disease modeling and toxicology screening. Looking ahead, the integration of next-generation sequencing, CRISPR/Cas9, and AI—including CNN-based vascularization monitoring and large language models (e.g., BioGPT-ER)—will drive precision diagnosis and personalized therapies for disorders like infertility and endometriosis, positioning 3D endometrial platforms at the forefront of regenerative reproductive medicine.

## 4. Discussions and Perspectives

Recent advances in endometrial stem cell biology and 3D culture technologies have significantly reshaped our understanding of cyclical regeneration, pathological remodeling, and embryo implantation. Technological advances in stem cell–derived assembloids and organ-on-chip platforms now enable unprecedented modeling of these complex interactions. By integrating patient-derived cells, biomechanical cues, and physiological hormone gradients, 3D models hold transformative potential not only for deciphering reproductive biology but also for developing targeted therapies for infertility and endometrial disorders.

Beyond mechanistic insights, organoids and assembloids are increasingly applied to pharmacological testing and drug delivery studies [126,127]. These platforms enable preclinical evaluation of anti-inflammatory and immunomodulatory agents, as well as localized delivery approaches—such as nanoparticles and hydrogels—that enhance drug penetration and minimize systemic toxicity [128]. Such strategies are particularly relevant to deep infiltrating endometriosis (DIE) [129], where poor vascularization limits the efficacy of systemic therapies and highlights the translational potential of organoid-based systems for personalized treatment. Nevertheless, significant challenges remain. Current organoids, assembloids, and microfluidic chips capture aspects of invasive growth and stromal–immune crosstalk but still fail to fully recapitulate the complexity of DIE, which involves extensive fibrosis, altered neuro–immune interactions, and resistance to hormonal therapy. Moreover, most models neglect circadian rhythm [130], a key regulator of immune recruitment, hormone responsiveness, and tissue remodeling [131,132]. Future systems that integrate rhythmic hormonal or mechanical cues within immune-competent microenvironments will be essential to enhance physiological relevance and predictive value.

Looking forward, integration of next-generation sequencing, machine learning, and CRISPR/Cas9 gene editing into 3D endometrial modeling is poised to transform the field. AI-guided optimization of organoid growth conditions, convolutional neural network-based monitoring of vascularization efficiency, and predictive modeling of implantation success under hormonal fluctuations are being actively developed. Furthermore, multimodal large language models (e.g., BioGPT-ER) are beginning to synthesize clinical and experimental data for personalized diagnosis and treatment of endometrial disorders such as infertility, recurrent implantation failure, and endometriosis. The convergence of bioengineering [133,134], stem cell biology [28,135], and computational modeling [136] will be critical for advancing regenerative medicine and reproductive precision therapies.

Ultimately, future efforts must focus on validating these models against clinical outcomes, incorporating immune-competent systems, and establishing regulatory-grade platforms for drug screening and personalized therapeutics. Multidisciplinary collaboration among reproductive biologists, bioengineers, data scientists, and clinicians will be essential to fully unlock the translational potential of stem cell-based endometrial reconstruction.

## Data Availability

No new data were created or analyzed in this study. Data sharing is not applicable.
